# Predicting AT(N) pathologies in Alzheimer’s disease from blood-based proteomic data using neural networks

**DOI:** 10.3389/fnagi.2022.1040001

**Published:** 2022-11-29

**Authors:** Yuting Zhang, Upamanyu Ghose, Noel J. Buckley, Sebastiaan Engelborghs, Kristel Sleegers, Giovanni B. Frisoni, Anders Wallin, Alberto Lleó, Julius Popp, Pablo Martinez-Lage, Cristina Legido-Quigley, Frederik Barkhof, Henrik Zetterberg, Pieter Jelle Visser, Lars Bertram, Simon Lovestone, Alejo J. Nevado-Holgado, Liu Shi

**Affiliations:** ^1^Department of Psychiatry, University of Oxford, Oxford, United Kingdom; ^2^Department of Biomedical Sciences, Reference Center for Biological Markers of Dementia (BIODEM), Institute Born-Bunge, University of Antwerp, Antwerp, Belgium; ^3^Department of Neurology, Universitair Ziekenhuis Brussel, Brussels, Belgium; ^4^Center for Neurociences (C4N), Vrije Universiteit Brussel, Brussels, Belgium; ^5^Complex Genetics Group, VIB Center for Molecular Neurology, Antwerp, Belgium; ^6^Department of Biomedical Sciences, University of Antwerp, Antwerp, Belgium; ^7^Department of Psychiatry, University of Geneva, Geneva, Switzerland; ^8^Department of Psychiatry and Neurochemistry, Institute of Neuroscience and Physiology, The Sahlgrenska Academy at the University of Gothenburg, Mölndal, Sweden; ^9^Centro de Investigación Biomédica en Red de Enfermedades Neurodegenerativas (CIBERNED), Hospital de la Santa Creu i Sant Pau, Barcelona, Spain; ^10^Department of Psychiatry, University Hospital of Lausanne, Lausanne, Switzerland; ^11^Department of Geriatric Psychiatry, University Hospital of Psychiatry and University of Zürich, Zürich, Switzerland; ^12^CITA-Alzheimer Foundation, San Sebastian, Spain; ^13^Kings College London, London, United Kingdom; ^14^The Systems Medicine Group, Steno Diabetes Center, Gentofte, Denmark; ^15^Department of Radiology and Nuclear Medicine, VU University Medical Center, Amsterdam, Netherlands; ^16^University College London (UCL) Institutes of Neurology and Healthcare Engineering, London, United Kingdom; ^17^Clinical Neurochemistry Laboratory, Sahlgrenska University Hospital, Mölndal, Sweden; ^18^UK Dementia Research Institute at UCL, London, United Kingdom; ^19^Department of Neurodegenerative Disease, UCL Institute of Neurology, London, United Kingdom; ^20^Hong Kong Center for Neurodegenerative Diseases, Hong Kong, China; ^21^Department of Psychiatry and Neuropsychology, School for Mental Health and Neuroscience, Alzheimer Centrum Limburg, Maastricht University, Maastricht, Netherlands; ^22^Alzheimer Center, VU University Medical Center, Amsterdam, Netherlands; ^23^Lübeck Interdisciplinary Platform for Genome Analytics, University of Lübeck, Lübeck, Germany; ^24^Department of Psychology, University of Oslo, Oslo, Norway; ^25^Janssen R&D, High Wycombe, United Kingdom

**Keywords:** Alzheimer’s disease, plasma proteomics, amyloid β, tau, neurodegeneration, machine learning, artificial neural networks

## Abstract

**Background and objective:**

Blood-based biomarkers represent a promising approach to help identify early Alzheimer’s disease (AD). Previous research has applied traditional machine learning (ML) to analyze plasma omics data and search for potential biomarkers, but the most modern ML methods based on deep learning has however been scarcely explored. In the current study, we aim to harness the power of state-of-the-art deep learning neural networks (NNs) to identify plasma proteins that predict amyloid, tau, and neurodegeneration (AT[N]) pathologies in AD.

**Methods:**

We measured 3,635 proteins using SOMAscan in 881 participants from the European Medical Information Framework for AD Multimodal Biomarker Discovery study (EMIF-AD MBD). Participants underwent measurements of brain amyloid β (Aβ) burden, phosphorylated tau (p-tau) burden, and total tau (t-tau) burden to determine their AT(N) statuses. We ranked proteins by their association with Aβ, p-tau, t-tau, and AT(N), and fed the top 100 proteins along with age and apolipoprotein E (*APOE*) status into NN classifiers as input features to predict these four outcomes relevant to AD. We compared NN performance of using proteins, age, and *APOE* genotype with performance of using age and *APOE* status alone to identify protein panels that optimally improved the prediction over these main risk factors. Proteins that improved the prediction for each outcome were aggregated and nominated for pathway enrichment and protein–protein interaction enrichment analysis.

**Results:**

Age and *APOE* alone predicted Aβ, p-tau, t-tau, and AT(N) burden with area under the curve (AUC) scores of 0.748, 0.662, 0.710, and 0.795. The addition of proteins significantly improved AUCs to 0.782, 0.674, 0.734, and 0.831, respectively. The identified proteins were enriched in five clusters of AD-associated pathways including human immunodeficiency virus 1 infection, p53 signaling pathway, and phosphoinositide-3-kinase–protein kinase B/Akt signaling pathway.

**Conclusion:**

Combined with age and *APOE* genotype, the proteins identified have the potential to serve as blood-based biomarkers for AD and await validation in future studies. While the NNs did not achieve better scores than the support vector machine model used in our previous study, their performances were likely limited by small sample size.

## Introduction

Alzheimer’s disease (AD) is a growing public health concern with no disease-modifying treatment available (Apostolova, 2016). The core criteria for clinical diagnosis of AD are based on behavioral and cognitive symptoms, but neuropathological changes in the central nervous system initiate years before the onset of cognitive impairment (Jack et al., 2013). The preclinical stage when pathologies develop in the absence of clinical symptoms presents an opportunity for early intervention with drugs to slow down or even halt disease progression. Currently, the well-established biomarkers for AD are cerebrospinal fluid (CSF) amyloid-β peptide (Aβ) and amyloid positron emission tomography (PET). They both highly correlate with pathologies found in brain autopsy (Strozyk et al., 2003; Curtis et al., 2015) and can identify early AD with high accuracy (Palmqvist et al., 2015). However, collecting CSF requires a lumbar puncture, an invasive practice that could lead to complications including post-dural puncture headache (de Almeida et al., 2011). PET imaging is expensive and requires specialist equipment that is not easily available. Thus, these two approaches have limited clinical utility. Blood-based biomarkers offer a desirable strategy to aid early diagnosis of AD. As blood-based test is minimally invasive, economical, and widely available, they can serve as efficient prescreening tools in a multi-stage diagnostic process (O’Bryant et al., 2016). Earlier research has provided evidence of alterations of proteomic profiles in blood samples associated with AD state (Hye et al., 2006; Ray et al., 2007), validating the feasibility of blood-based biomarkers.

The development of blood-based biomarkers is facilitated by advances in not only targeted approaches (e.g., plasma phosphorylated tau measurements; Ashton et al., 2021; Milà-Alomà et al., 2022) but also untargeted large-scale omics technologies. Researchers have adopted high-resolution mass spectrometry for proteomic profiling of blood and discovery of AD protein signatures (Lopez et al., 2005; Dey et al., 2019). Highly sensitive multiplexed immunoassay platforms, such as Olink (Whelan et al., 2019; Jiang et al., 2022), and aptamer-based assay platforms, such as SOMAscan (Kiddle et al., 2014; Sattlecker et al., 2014), have further allowed researchers to capture the complexity of plasma proteome and identify prospective biomarkers by measuring thousands of proteins simultaneously in thousands of individuals with a single platform. In analyzing the rich omics data, machine learning (ML), a subdomain of artificial intelligence, has proven an invaluable tool (Li et al., 2021). Previous studies have had great success in finding blood analytes that predict AD-related measures using traditional ML algorithms such as support vector machines (Voyle et al., 2015; Shi L. et al., 2019; Karaglani et al., 2020), decision trees (Pérez-Grijalba et al., 2019; Shi Y. et al., 2019), and random forests (Goudey et al., 2019; Beltrán et al., 2020; Lin et al., 2020; Zhao et al., 2020).

Deep learning neural networks (NNs) are the last iteration of ML methods and have significant advantages when compared to the older ML methods both in terms of classification accuracy and versatility, yet fewer studies have explored their utility in blood-based AD biomarker discovery. As they are capable of learning data representations in multiple hierarchies, they have often outperformed conventional models in various applications including predicting clinical diagnoses (Durstewitz et al., 2019). Thus, in the current study, we implement deep NNs to analyze plasma proteomics measured by SOMAscan. Our objective is to identify candidate plasma protein panels to detect amyloid, tau, and neurodegeneration (AT[N]) pathologies in AD.

## Materials and methods

### Participants

European Medical Information Framework (EMIF, www.emif.eu) is funded by the Innovative Medicines Initiative to support the reuse of existing healthcare data. As part of this project, EMIF-AD set up the Multimodal Biomarker Discovery (MBD) study by integrating multi-omics data to facilitate the development of AD biomarkers. Participants of the current study were from the EMIF 1000 sub-cohort assembled in a previous study (Bos et al., 2018). The original sub-cohort included 1,221 participants from 11 single- or multi-center studies across Europe. In the current study, we included only subjects who had available plasma samples, resulting in a subset of 881 participants from 10 studies. Among them, 311 had normal cognition (CN), 386 had mild cognitive impairment (MCI), and 184 had a diagnosis of AD dementia.

All 881 participants underwent measurement of the concentration of the 42 amino acid-long Aβ protein, Aβ_42_, in CSF, sometimes in a ratio with the 40 amino acid-long form, Aβ_40_, or amyloid PET as the primary AD outcome. A previous study has classified the amyloid burden of each participant as high or low based on z-score cutoffs (Bos et al., 2018). In addition, a subgroup of 787 subjects underwent measurement of CSF phosphorylated tau (p-tau), and a subgroup of 791 subjects underwent measurement of CSF total tau (t-tau). CSF p-tau and t-tau levels were both measured locally, and their statuses were classified as high or low with local cutoffs. Mini-mental state examination (MMSE) was administered to a majority of participants to assess cognitive function. All participants were genotyped to determine whether they were apolipoprotein E (*APOE*) risk allele ε4 carriers or non-carriers. Lastly, proteins in plasma samples were measured by the SOMAscan assay (SomaLogic Inc.) in a previous study (Shi L. et al., 2019). SOMAscan is an aptamer-based platform which transforms individual protein signals into corresponding chemically-modified nucleotide signals that can be quantified by relative fluorescence on DNA microarrays (Gold et al., 2010). Plasma samples were divided into two groups, and each group was processed independently. Forty samples were tested in both batches to normalize the measurements across assay runs. A total of 3,635 plasma proteins were quantified.

### Statistical analysis

All statistical analyses were performed using Python (version 3.9.7). We compared the demographic and clinical characteristics of patients with different diagnoses. Continuous variables were compared between groups by the Kruskal-Wallis one-way ANOVA test followed by Mann–Whitney *U*-tests for pairwise comparisons. Categorical variables were compared between groups by Chi-square test.

### Building NNs

**Figure 1:**
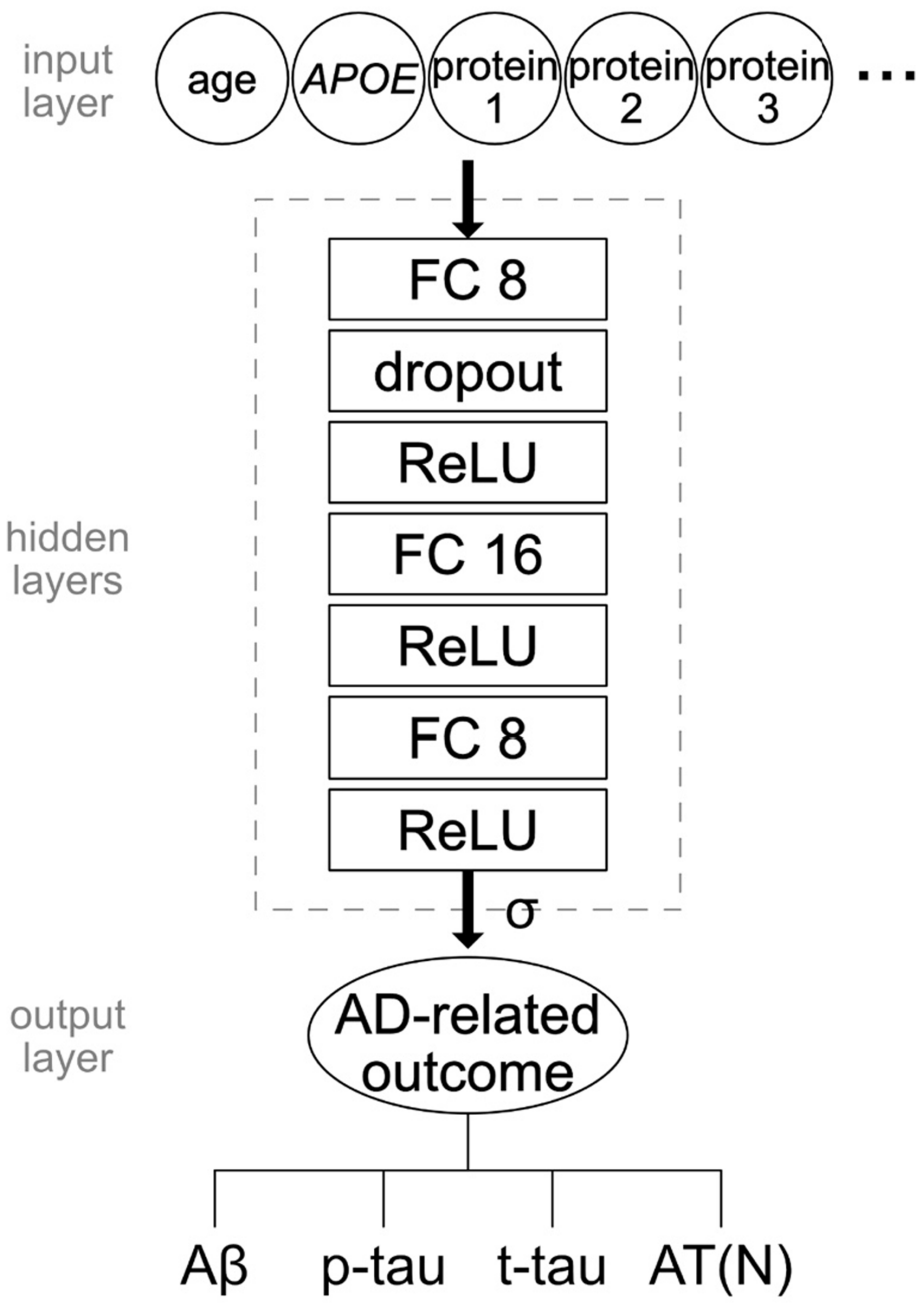
Structure of the NN model. The dimension of the input layer was determined by the number of features including age, *APOE* status, and/or protein levels. The hidden layers consisted of three fully connected layers of size 8, 16, and 8 connected by ReLU activation function. Dropout of rate 0.5 was applied to the output of the first hidden layer. The final output was transformed by a sigmoid function. Four AD-related measures were independently tested as outcomes: Aβ, p-tau, t-tau, and AT(N).

NNs were implemented using the Python package PyTorch (version 1.10.1). The network (Figure 1) consisted of three fully connected hidden layers with sizes 8, 16, and 8. Dimension of the input layer varied depending on the number of features: one for age, one for *APOE* status, and one for each protein. The output layer had one dimension to encode the binary outcome. Dropout was applied to the output of the first hidden layer at a rate of 0.5. Each hidden layer was followed by a rectified linear unit (ReLU) activation function. The loss function was binary cross-entropy with sigmoid. Learning was implemented using the Adam stochastic optimization algorithm with a learning rate of 0.01.

Training and testing were implemented with 5-fold cross-validation. The dataset was shuffled across samples before splitting between training and testing. On each training/testing split, the parameters of the NN (i.e., weights) were reinitialized, and the NN was trained for 15 epochs. In each epoch, data was fed into NN in minibatches of size 128. The outputs were transformed by a sigmoid function to compare with the binary ground-truth values.

### Discrimination of AT(N)

The dataset was preprocessed for analysis. Age was power-transformed and z-scored. All protein levels were power-transformed and z-scored to have zero mean and unit variance. The effects of study and blood freeze–thaw cycles on proteins were removed by linear regression, and the resulting residuals replaced raw protein levels.

We aim to predict Aβ burden (high vs. low), p-tau burden (high vs. low), t-tau burden (high vs. low), and AT(N) profile (high Aβ/p-tau/t-tau vs. low Aβ/p-tau/t-tau) from plasma proteins along with age and *APOE* genotype. For each of these classification objectives, we first performed logistic regression analysis to measure the linear association between each protein and the target and ranked the proteins by ascending *p* values. We then selected from 1 to 100 top-ranked proteins along with age and *APOE* status as input features for NN classification and compared their performance with that of age and *APOE* ε4 status alone. For each unique set of features and the target, we repeated training–testing 10 times to obtain the average receiver operating characteristic (ROC) curve and the area under the curve (AUC). AUC ROC scores resulting from different features were compared using independent t-tests.

### Enrichment analysis

The proteins that achieved the best performance in NN classification along with age and *APOE* status in differentiating high and low Aβ, p-tau, t-tau, and AT(N) were aggregated and nominated for pathway enrichment and protein–protein interaction enrichment analysis. The analysis was performed using the Metascape software (Zhou et al., 2019). The complete set of proteins was provided as “background.” The inputs were searched against KEGG Pathway database for pathway enrichment analysis and STRING, BioGrid, OmniPath, and InWeb_IM databases for protein–protein interaction analysis.

## Results

### Demographic and clinical variables

The current study included 881 participants from the EMIF 1000 sub-cohort. The demographic and clinical variables for each diagnostic group are described in Table 1. Patients with AD or MCI were older than CN subjects (AD vs. CN: odds ratio [OR] = 1.08, *p* < 0.001; MCI vs. CN: OR = 1.08, *p* < 0.001). CN subjects had higher MMSE scores than MCI subjects (OR = 2.08, *p* < 0.001), and MCI subjects had higher MMSE scores than AD subjects (OR = 1.44, *p* < 0.001). AD patients also had a higher prevalence of *APOE* ε4 carriers (AD vs. MCI: OR = 1.67, *p* < 0.001; AD vs. CN: OR = 2.54, *p* < 0.01). There was no statistical difference in sex distribution between diagnostic groups (AD vs. MCI: OR = 1.01, *p* = 1.00; MCI vs. CN: OR = 0.85, *p* = 0.31; AD vs. CN: OR = 1.01, *p* = 0.44). For AT(N) biomarkers, AD subjects had a higher prevalence of low CSF Aβ_42_ or Aβ_42/40_ or positive amyloid PET, high CSF p-tau, and high CSF t-tau than MCI subjects (Aβ: OR = 5.00; p-tau: OR = 2.03; t-tau: OR = 3.23; all *p* < 0.001) and CN subjects (Aβ: OR = 20.36; p-tau: OR = 9.69; t-tau: OR = 17.43; all *p* < 0.001).

**Table 1:** Demographic and clinical characteristics of the study population.

Characteristic	Sample size	CN	MCI	AD	*P-*value
*N*	881	311	386	184	
Age, median (IQR)	881	66.0 (58.9–70.0)	70.5 (65.1–75.5)	71.0 (63.4–77.0)	<0.001^*^
Male sex, *N* (%)	881	133 (42.8%)	181 (46.9%)	86 (46.7%)	0.509
MMSE, median (IQR)	878	29.0 (28.0–30.0)	26.5 (25.0–28.0)	22.0 (18.0–25.0)	<0.001^*^
*APOE* ε4+, *N* (%)	881	118 (37.9%)	186 (48.2%)	112 (60.9%)	<0.001^*^
Aβ+, *N* (%)	881	93 (30.0%)	245 (63.5%)	165 (89.7%)	<0.001^*^
P-tau+, *N* (%)	787	42 (19.1%)	203 (53.0%)	128 (69.6%)	<0.001^*^
T-tau+, *N* (%)	791	45 (20.1%)	221 (57.6%)	149 (81.4%)	<0.001^*^

CN, normal cognition; MCI, mild cognitive impairment; AD, Alzheimer’s disease; IQR, interquartile range; MMSE, mini-mental state examination; APOE, apolipoprotein E; Aβ, amyloid-β; p-tau, phosphorylated-tau; t-tau, total tau.

* *p* < 0.05.

### Discrimination of AT(N) markers using NNs

**Figure 2:**
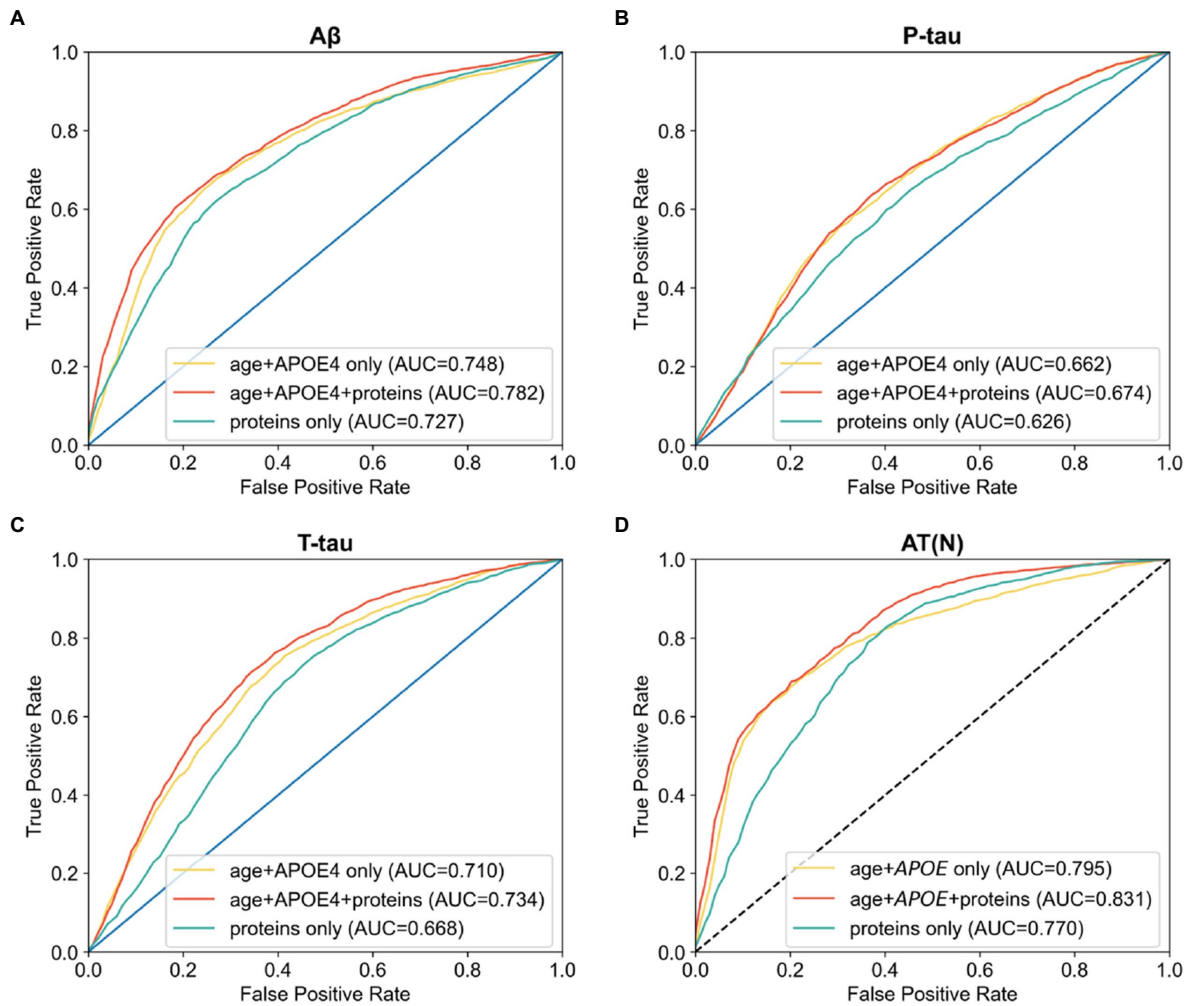
AUC ROCs of using age and *APOE* alone, age and *APOE* with proteins, and proteins alone to differentiate high vs. low **(A)** Aβ, **(B)** p-tau, **(C)** t-tau, and **(D)** combined AT(N) burden. For each target, the panel of proteins with the best performance were selected.

We first used regression analysis to find the linear association between each protein and Aβ. Binary Aβ burden (high: *N* = 503; low: *N* = 378) was defined by z-score cutoff of CSF Aβ_42/40_ at 0.061 or local cutoffs of CSF Aβ_42_ and amyloid PET. Out of all proteins, 1,793 reached statistical significance (uncorrected *p* < 0.05), and 1,492 of them reached the false discovery rate after correction for multiple comparisons (corrected *p* < 0.05). We then sought to find the optimal set of proteins that differentiate high vs. low Aβ burden using NNs. The combination of age and *APOE* ε4 alone achieved an AUC of 0.748 (95% confidence interval [CI] 0.745–0.750). With protein features alone, a panel of 15 proteins achieved the highest AUC of 0.727 (95% CI 0.722–0.732). After the combination of age, *APOE* ε4, and proteins as input features, we found a panel of 11 proteins (see Supplementary Table 1) that achieved the highest predictive value with an AUC of 0.782 (95% CI 0.779–0.785). This was significantly higher than AUCs from using only age and *APOE* ε4 status (*p* < 0.001; Figure 2A).

Binary p-tau burden (high: *N* = 373; low: *N* = 414) was defined by local cutoffs of CSF p-tau levels. For p-tau, 690 proteins reached statistical significance (uncorrected *p* < 0.05) while none passed the false discovery rate (corrected *p* < 0.05). In classifying high and low p-tau, age and *APOE* ε4 alone achieved an AUC of 0.662 (95% CI 0.658–0.666). With protein features alone, the set of 99 proteins achieved the highest AUC of 0.626 (95% CI 0.615–0.636). After the combination of age, *APOE* ε4, and proteins, we found that the addition of 2 proteins (see Supplementary Table 1) achieved the highest AUC of 0.674 (95% CI 0.669–0.678), which was significantly greater than the AUCs from age and *APOE* alone (*p* < 0.001; Figure 2B).

Binary t-tau burden (high: *N* = 415; low: *N* = 376) was defined by local cutoffs of CSF t-tau levels. For t-tau, 1,437 out of all proteins reached statistical significance (uncorrected *p* < 0.05), and 1,038 of them reached the false discovery rate (corrected *p* < 0.05). Age and *APOE* ε4 together achieved an AUC of 0.710 (95% CI 0.708–0.713). With protein features alone, a panel of 33 proteins achieved the highest AUC of 0.668 (95% CI 0.660–0.675). Combining with age and *APOE* ε4, a panel of 29 proteins (see Supplementary Table 1) achieved the highest AUC of 0.734 (95% CI 0.729–0.738), significantly exceeding that of age and *APOE* alone (*p* < 0.001; Figure 2C).

### Discrimination of A + T + N+ from A – T – N − using NNs

We further used proteins to differentiate subjects with extreme AT(N) profiles using NNs. High and low AT(N) levels (A + T + N+: *N* = 298; A − T − N−: *N* = 229) were defined using the cutoffs of binary Aβ, p-tau, and t-tau burden. In logistic regression analysis, 1,638 proteins reached statistical significance (uncorrected *p* < 0.05) in association with A + T + N+ and A − T − N−. 1,317 of them passed the false discovery rate (corrected *p* < 0.05). The correlations of protein *p* values between each pair of AD-related outcomes are listed in Supplementary Table 2.

In differentiating A + T + N+ and A − T − N−, age and *APOE* ε4 alone achieved an AUC of 0.795 (95% CI 0.793–0.798). Using the top 100 proteins as input features, we found that 86 proteins achieved the highest AUC of 0.770 (95% CI 0.765–0.774). The combination of age, *APOE* ε4, and proteins showed that a panel of 7 proteins (see Supplementary Table 1) reached the highest AUC of 0.831 (95% CI 0.827–0.835), which was significantly better than those obtained from age and *APOE* ε4 alone (*p* < 0.05; Figure 2D). All NN classification results are summarized in Table 2. NN performance scores of using from 1 to 100 top proteins with or without age and *APOE* status to classify each outcome are shown in Supplementary Figure 1.

**Table 2:** Summary of AUC scores (95% CI) of NN classification.

	Number of subjects (high/low)	Age + *APOE* only	Proteins only	Proteins + age + *APOE*
Aβ	503/378	0.748 (0.745–0.750)	0.727 (0.722–0.732)	0.782 (0.779–0.785)
P-tau	373/414	0.662 (0.658–0.666)	0.626 (0.615–0.636)	0.674 (0.669–0.678)
T-tau	415/376	0.710 (0.708–0.713)	0.668 (0.660–0.675)	0.734 (0.729–0.738)
AT(N)	298/229	0.795 (0.793–0.798)	0.770 (0.765–0.774)	0.831 (0.827–0.835)

AUC, area under the receiver operating characteristic curve; CI, confidence interval; NN, neural networks; APOE, apolipoprotein E; Aβ, amyloid-β; p-tau, phosphorylated-tau; t-tau, total tau; AT(N), amyloid/tau/neurodegeneration.

### Enriched terms

Aggregately, 35 proteins were nominated for the pathway enrichment analysis. They were matched to 34 unique genes. The background list consisted of 3,306 unique proteins in UniProt identifiers, which were matched to 3,268 genes. A total of 11 pathways were identified (Table 3). They were grouped into five clusters based on their membership similarities. Each cluster was represented by its most significant pathway: human immunodeficiency virus 1 infection (uncorrected *p* < 0.001), p53 signaling pathway (uncorrected *p* = 0.001), phosphoinositide-3-kinase–protein kinase B/Akt (PI3K-Akt) signaling pathway (uncorrected *p* = 0.009), complement and coagulation cascades (uncorrected *p* = 0.018), and mitogen-activated protein kinase (MAPK) signaling pathway (uncorrected *p* = 0.045). The enriched pathways were not significant after correction for multiple comparisons (corrected *p* > 0.05). The protein–protein interaction analysis revealed three significant terms (Table 4): PI3K-Akt signaling pathway (*p* < 0.001), complement and coagulation cascades (*p* < 0.001), and proteoglycans in cancer (*p* < 0.005).

**Table 3:** Pathway enrichment analysis revealed 11 significantly enriched pathways.

Term ID	Description	*P-*value (uncorrected)	*P-*value (corrected)
hsa05170	Human immunodeficiency virus 1 infection	< 0.001	0.203
hsa04218	Cellular senescence	0.009	0.641
hsa05145	Toxoplasmosis	0.015	0.754
hsa05014	Amyotrophic lateral sclerosis	0.036	1.000
hsa04115	p53 signaling pathway	0.001	0.209
hsa04114	Oocyte meiosis	0.005	0.524
hsa04151	PI3K-Akt signaling pathway	0.009	0.641
hsa05202	Transcriptional misregulation in cancer	0.013	0.747
hsa04610	Complement and coagulation cascades	0.018	0.770
hsa04936	Alcoholic liver disease	0.029	1.000
hsa04010	MAPK signaling pathway	0.045	1.000

They are grouped into five clusters based on membership similarities. PI3K-Akt, phosphoinositide-3-kinase–protein kinase B/Akt; MAPK, mitogen-activated protein kinase.

**Table 4:** Protein–protein interaction enrichment analysis revealed three significantly enriched terms.

Term ID	Description	*P-*value
hsa04151	PI3K-Akt signaling pathway	<0.001
hsa04610	Complement and coagulation cascades	<0.001
hsa05205	Proteoglycans in cancer	<0.005

PI3K-Akt, phosphoinositide-3-kinase–protein kinase B/Akt.

## Discussion

In this study, we used regression analysis and NNs to obtain the optimal sets of protein features that discriminated high and low AT(N) burdens in AD. Age and *APOE* ε4 status alone achieved an AUC of 0.748 in predicting Aβ, an AUC of 0.662 in predicting p-tau, an AUC of 0.710 in predicting t-tau, and an AUC of 0.795 in predicting AT(N) abnormality. The addition of proteins significantly improved prediction of Aβ (AUC = 0.782), p-tau (AUC = 0.674), t-tau (AUC = 0.734) and AT(N) profiles (AUC = 0.831).

We selected the variables age and *APOE* genotype for comparison as advanced age and presence of the *APOE* ε4 allele are two of the strongest risk factors for AD (Riedel et al., 2016). In addition, our previous study confirmed that among age, sex, education, and *APOE* genotype, the combination of these two characteristics best predicts Aβ pathologies in the current cohort (Shi Y. et al., 2019). The previous study also classified amyloid burden using plasma proteomics and obtained similar results: the combination of age, *APOE* ε4 status, and proteins achieved the highest AUC score of 0.78, outperforming demographic variables alone. But as different protein ranking methods were employed, our panel of 11 proteins predicting Aβ has little overlap with the previously identified 44 proteins and provides novel candidates for validation. In the previous study, top proteins were selected through Lasso which uses a regularization technique to choose features that correlate with the outcome but not with each other, thus reducing redundant inputs. While this method can enhance prediction accuracy in an independent testing set, it may exclude proteins that have linearly redundant association with the outcome but nonlinear effects that can be detected by neural networks. As logistic regression does not penalize feature collinearity, the proteins selected using this method may retain meaningful information for neural network training. Their performance corroborates the utility of plasma proteomics in predicting Aβ pathology demonstrated in other recent studies (Ashton et al., 2019; Park et al., 2019; Westwood et al., 2020). Considering that AD is a complex disorder with mixed pathologies, we further evaluated the potential of plasma proteomics in predicting the other two components of the AT(N) framework. In accordance with previous findings (Shi et al., 2021), our results suggest that blood-based protein panels can reflect brain tau burden and neurodegeneration in addition to Aβ abnormality. Finally, a panel of proteins showed satisfactory performance in predicting combined AT(N) profiles, supporting the potential of plasma proteomics to act as comprehensive biomarkers for core AD pathologies. Interestingly, the statistical significance measures of the association between proteins and each AD-related outcomes have high correlations, suggesting that these pathological features are closely related, which is consistent with our previous finding showing that Aβ has a causal relationship with tau pathology (Shi L. et al., 2019).

We performed a pathway enrichment analysis on proteins in identified panels and found 5 clusters of pathways. One of them is the complement and coagulation cascades, which have been reported previously in a systematic review of blood-based protein biomarkers (Kiddle et al., 2014). Neuroinflammation is implicated as having substantial involvement in the pathogenesis of AD (Heneka et al., 2015). As part of the innate immune system, the complement system is activated by Aβ deposits and in turn damages the neurons *via* self-attack (McGeer and McGeer, 2002). Its activation is accompanied by an upregulation of coagulation factors (Amara et al., 2010) associated with the neurovascular damages observed in AD brain. A previous study supports the association between the peripheral activation of this pathway and AD-specific pathologies (Pillai et al., 2019). Notably, the complement protein C4 was nominated in multiple panels. A previous study has found that C4 could discriminate rapidly and slowly progressing AD (Thambisetty et al., 2010), suggesting that it might be indicative of AD severity and is a potentially promising biomarker for early stages of AD. Another significant pathway identified is the MAPK pathway, which has been recognized in the AlzPathway, a comprehensive map of pathways related to AD (Mizuno et al., 2012). In AD, activation of the serine/threonine MAPK in response to extracellular stimuli promotes neuronal apoptosis (Munoz and Ammit, 2010). C-Jun N-terminal kinases (JNK) and p38, two members of the MAPK family, are involved in the hyperphosphorylation of tau (Goedert et al., 1997; Reynolds et al., 2000). JNK is also thought to regulate the phosphorylation and degradation of amyloid precursor proteins (Muresan and Muresan, 2007; Colombo et al., 2009). Our results provide further evidence for the dysregulation of the MAPK cascade proteins in AD.

This study leveraged the advancement of ML. While traditional ML models have been predominant in previous investigation of AD biomarkers, deep NNs are expected to play a more significant role moving forward. They are capable of detecting complex nonlinear patterns in raw data and are highly sensitive to the relevance of information received (LeCun et al., 2015). Recent studies have demonstrated the superior performance of deep learning in detecting AD disease stage and predicting longitudinal progression of AD using multimodal information (Venugopalan et al., 2021; El-Sappagh et al., 2022). In agreement, our study indicates the great potential of deep learning approaches to capture the complexity of blood-based omics data and facilitate the discovery of candidate biomarkers. While the performance of NNs in this study did not exceed that of the support vector machine used in our previous study (Shi L. et al., 2019), this might result from scarcity of data as training of deep learning models typically benefits from extremely large sample sizes (Ellis and Morgan, 1999). A similar study found that the more conventional random forests outperformed deep learning models in differentiating AD from CN using plasma metabolomics and reached the same conclusion (Stamate et al., 2019). While NN behavior has been much less explored in bioinformatic data, extensive deep learning research in imaging, video, audio, and natural language processing has consistently shown that model performance increases with data size, a phenomenon now known as the scaling laws (Brown et al., 2020; Wei et al., 2022). In light of these observations, it is plausible that a similar effect would become apparent in omics as data sizes grow orders of magnitude larger than thousands of samples still typically used in present-day studies. Model architecture should also be considered in the future development of AD biomarkers. More complex NNs can be adopted to take advantage of an increasing quantity of heterogeneous clinical and biological data. Recently, TabNet has been proposed as a NN architecture specializing in tabular data processing by applying sequential attention to select the best features at each decision step (Arık and Pfister, 2021). It would be interesting to see whether innovative models like TabNet could be applied to explore the predictive power of putative biomarkers and integrated into the biomarker development pipeline.

One limitation of the current study is that no data was withheld for validation of the effect of the nominated proteins. However, this helps minimize overfitting and optimize the generalizability of those proteins at the discovery stage. To assess their applicability in the larger population, future validation using independent cohorts is preferred. In addition, it is important to note that participants with AD were significantly older than MCI or CN participants in this study, so age and age-related changes in plasma profiles might contribute to most of the predictive accuracy. As development of AD is not always associated with age in the larger population, it is important to test the validity of candidate biomarkers among patients and controls of similar ages.

In conclusion, the opportunity of the clinical implementation of blood-based biomarkers for AD is exciting. The current study supports the use of proteomics measured by SOMAscan for the discovery blood-based biomarkers. In addition, NNs show great utility in predicting disease pathologies from proteomics which encourages the adoption of more advanced ML approaches in future investigation. Using these state-of-the-art technologies, we identified several proteins that are involved in AD-related pathways and can potentially serve as prescreening tools for the early detection of AD-specific pathologies when combined with demographic information.

## Data availability statement

The data analyzed in this study is subject to the following licenses/restrictions: the dataset analyzed for this study is available upon request *via* the EMIF-AD Catalog (https://emif-catalogue.eu) after approval of the research question by all parent cohorts and the EMIF-AD team. Requests to access these datasets should be directed to EMIF-AD, https://emif-catalogue.eu.

## Ethics statement

The studies involving human participants were reviewed and approved by Aristotle University of Thessaloniki Medical School Ethics Committee; Ethics Committee of the Medical Faculty Mannheim, University of Heidelberg; Ethic and Clinical Research Committee Donostia; Ethics Committee Inserm and Aix Marseille University; The Healthcare Ethics Committee of the Hospital Clínic; Central Clinical Research and Clinical Trials Unit (UICEC Sant Pau); INSERM Ethical Committee; Ethic Committee of the IRCCS San Giovanni di Dio FBF; Comitato Etico IRCCS Pascale - Napoli; Ethics Committee at Karolinska Institutet; Ethische commissie onderzoek UZ/KU Leuven; Research Ethics Committee Lausanne University Hospital; Medical Ethical Committee Maastricht University Medical Center; Committee on Health Research Ethics, Region of Denmark; Ethics Committee of Mediterranean University; University of Lille Ethics Committee; Ethical Committee at the Medical Faculty, Leipzig University; Ethical Committee at the Medical Faculty, University Hospital Essen; Ethics Committee University of Antwerp; Ethical Committee of University of Genoa; Ethics Committee, University of Gothenburg; Human Ethics Committee of the University of Perugia; and Medical Ethics Committee VU Medical Center. The patients/participants provided their written informed consent to participate in this study.

## Author contributions

AN-H, LS, and YZ contributed to conception and design of the study. YZ analyzed the data, interpreted the results, and drafted and revised the manuscript. All authors contributed to the article and approved the submitted version.

## Funding

This research was conducted as part of the EMIF-AD project which has received support from the Innovative Medicines Initiative Joint Undertaking under EMIF grant agreement no. 115372, resources of which are composed of financial contribution from the European Union’s Seventh Framework Programme (FP7/2007–2013) and EFPIA companies’ in-kind contribution. The authors declare that they have received funding from Astra Zeneca (SL) and Janssen (SL and ANH). The funders were not involved in the study design, collection, analysis, interpretation of data, the writing of this article, or the decision to submit it for publication. The DESCRIPA study was funded by the European Commission within the 5th framework program (QLRT-2001-2455). The EDAR study was funded by the European Commission within the 5th framework program (contract # 37670). The San Sebastian GAP study was partially funded by the Department of Health of the Basque Government (allocation 17.0.1.08.12.0000.2.454.01.41142.001.H). The research at VIB-CMN was funded in part by the University of Antwerp Research Fund. LS is funded by the Virtual Brain Cloud from European commission (grant no. H2020-SC1-DTH-2018-1). HZ is a Wallenberg Scholar supported by grants from the Swedish Research Council (#2018–02532), the European Research Council (#681712 and #101053962), Swedish State Support for Clinical Research (#ALFGBG-71320), the Alzheimer Drug Discovery Foundation (ADDF), United States (#201809–2016862), the AD Strategic Fund and the Alzheimer’s Association (#ADSF-21-831376-C, #ADSF-21-831381-C, and #ADSF-21-831377-C), the Bluefield Project, the Olav Thon Foundation, the Erling-Persson Family Foundation, Stiftelsen för Gamla Tjänarinnor, Hjärnfonden, Sweden (#FO2022-0270), the European Union’s Horizon 2020 research and innovation programme under the Marie Skłodowska-Curie grant agreement no 860197 (MIRIADE), the European Union Joint Programme – Neurodegenerative Disease Research (JPND2021-00694), the UK Dementia Research Institute at UCL (UKDRI-1003), and the Lausanne cohort was supported by grants from the Swiss National Science Foundation (SNF 320030_141179), Synapsis Foundation – Dementia Research Switzerland (grant no. 2017-PI01). This work was supported by the Centre for Artificial Intelligence in Precision Medicines of the University of Oxford and King Abdulaziz University.

## Conflict of interest

SL is named as an inventor on biomarker intellectual property protected by Proteome Sciences and Kings College London unrelated to the current study and within the past 5 years has advised for Optum labs, Merck, SomaLogic and been the recipient of funding from AstraZeneca and other companies *via* the IMI funding scheme. SL is employed by company Janssen. HZ has served at scientific advisory boards and/or as a consultant for Abbvie, Acumen, Alector, ALZPath, Annexon, Apellis, Artery Therapeutics, AZTherapies, CogRx, Denali, Eisai, Nervgen, Novo Nordisk, Passage Bio, Pinteon Therapeutics, Red Abbey Labs, reMYND, Roche, Samumed, Siemens Healthineers, Triplet Therapeutics, and Wave, has given lectures in symposia sponsored by Cellectricon, Fujirebio, Alzecure, Biogen, and Roche, and is a co-founder of Brain Biomarker Solutions in Gothenburg AB (BBS), which is a part of the GU Ventures Incubator Program, all unrelated to this study. AL has served at scientific advisory boards of Biogen, Fujirebio Europe, Eli Lilly, Grifols, Novartis, Nutricia, Roche, and Otsuka and is the inventor of a patent on synaptic markers in CSF, all unrelated to this study. JP has served at scientific advisory boards of Fujirebio Europe, Eli Lilly, and Nestlé Institute of Health Sciences, all unrelated to this study. SE has received unrestricted research grants from Janssen Pharmaceutica and ADx Neurosciences and has served at scientific advisory boards of Biogen, Eisai, Novartis, Nutricia/Danone, and Roche, all unrelated to this study.

The remaining authors declare that the research was conducted in the absence of any commercial or financial relationships that could be construed as a potential conflict of interest.

## Publisher’s note

All claims expressed in this article are solely those of the authors and do not necessarily represent those of their affiliated organizations, or those of the publisher, the editors and the reviewers. Any product that may be evaluated in this article, or claim that may be made by its manufacturer, is not guaranteed or endorsed by the publisher.
