# Supplementary material for: Predicting AT(N) pathologies in Alzheimer’s disease from blood-based proteomic data using neural networks
Source: Front Aging Neurosci. 2022 Nov 29;14:1040001. doi: 10.3389/fnagi.2022.1040001 (PMC9746615; doi:10.3389/fnagi.2022.1040001)

Supplementary Material

Supplementary Table 1. Four panels of proteins of sizes 11, 2, 29, and 7 respectively resulted in the best performance in NN classification of high vs. low Aβ, p-tau, t-tau, and AT(N) burden when combined with age and *APOE* ε4 status. These four panels were listed below. In each panel, the proteins were sorted by corrected *p* values from the regression analysis.

UniProt	Protein Name
**Aβ**	
O75437	Tubulin-specific chaperone A
Q6UXK5	Leucine-rich repeat neuronal protein 1
P0C0L4/P0C0L5	Complement C4-A/B
O00391	Sulfhydryl oxidase 1
Q13113	PDZK1-interacting protein 1
P02775	Platelet basic protein
Q92765	Secreted frizzled-related protein 3
P02751	Fibronectin
P63098	Calcineurin subunit B type 1
Q9H2E6	Semaphorin-6A
P04004	Vitronectin
**P-tau**	
O75347	Tubulin-specific chaperone A
P14635	G2/mitotic-specific cyclin-B1
**T-tau**	
P0C0L4/P0C0L5	Complement C4-A/B
P08294	Extracellular superoxide dismutase
O75347	Tubulin-specific chaperone A
P02775	Platelet basic protein

Q9H2E6	Semaphorin-6A
Q96PQ1	Sialic acid-binding Ig-like lectin 12
Q07817	Bcl-2-like protein 1
Q9P109	Beta-1,3-galactosyl-O-glycosyl-glycoprotein beta-1,6-N-acetylglucosaminyltransferase 4
Q96KP4	Cytosolic non-specific dipeptidase
Q96A84	EMI domain-containing protein 1
P48736	Phosphatidylinositol 4,5-bisphosphate 3-kinase catalytic subunit gamma isoform
Q9NRN5	Olfactomedin-like protein 3
P07202	Thyroid peroxidase
Q6UX15	Layilin
P30101	Protein disulfide-isomerase A3
Q9BY41	Histone deacetylase 8
Q14982	Opioid-binding protein/cell adhesion molecule
O60869	Endothelial differentiation-related factor 1
P63098	Calcineurin subunit B type 1
Q9P0G3	Kallikrein-14
Q92765	Secreted frizzled-related protein 3
O75351	Vacuolar protein sorting-associated protein 4B
Q8NCW0	Kremen protein 2
P05019	Insulin-like growth factor I
O00391	Sulfhydryl oxidase 1
P07951	Tropomyosin beta chain
P46734	Dual specificity mitogen-activated protein kinase kinase 3
Q08431	Lactadherin
P36888	Receptor-type tyrosine-protein kinase FLT3

**AT(N)**	
O75347	Tubulin-specific chaperone A
Q6UXK5	Leucine-rich repeat neuronal protein 1
P02775	Platelet basic protein
Q9H2E6	Semaphorin-6A
P0C0L4/P0C0L5	Complement C4-A/B
Q96A84	EMI domain-containing protein 1
O00391	Sulfhydryl oxidase 1

Supplementary Table 2. Spearman's rank correlation coefficients between protein *p* values obtained from logistic regression with each AD-related outcome.

	**AT(N)**	**T-tau**	**P-tau**
**Aβ**	0.887	0.736	0.587
**P-tau**	0.776	0.712	
**T-tau**	0.892		

Supplementary Figure 1. We implemented NNs to predict high vs. low **(A)** Aβ, **(B)** p-tau, **(C)** t-tau, and **(D)** AT(N) burden from proteins, age, and *APOE* ε4 status. From 1 to 100 top proteins, sorted by their significance of linear association with the AD-related outcome, were used as input features. For each combination of input and output, NNs were trained and tested 10 times to obtain the average AUC ROC scores.

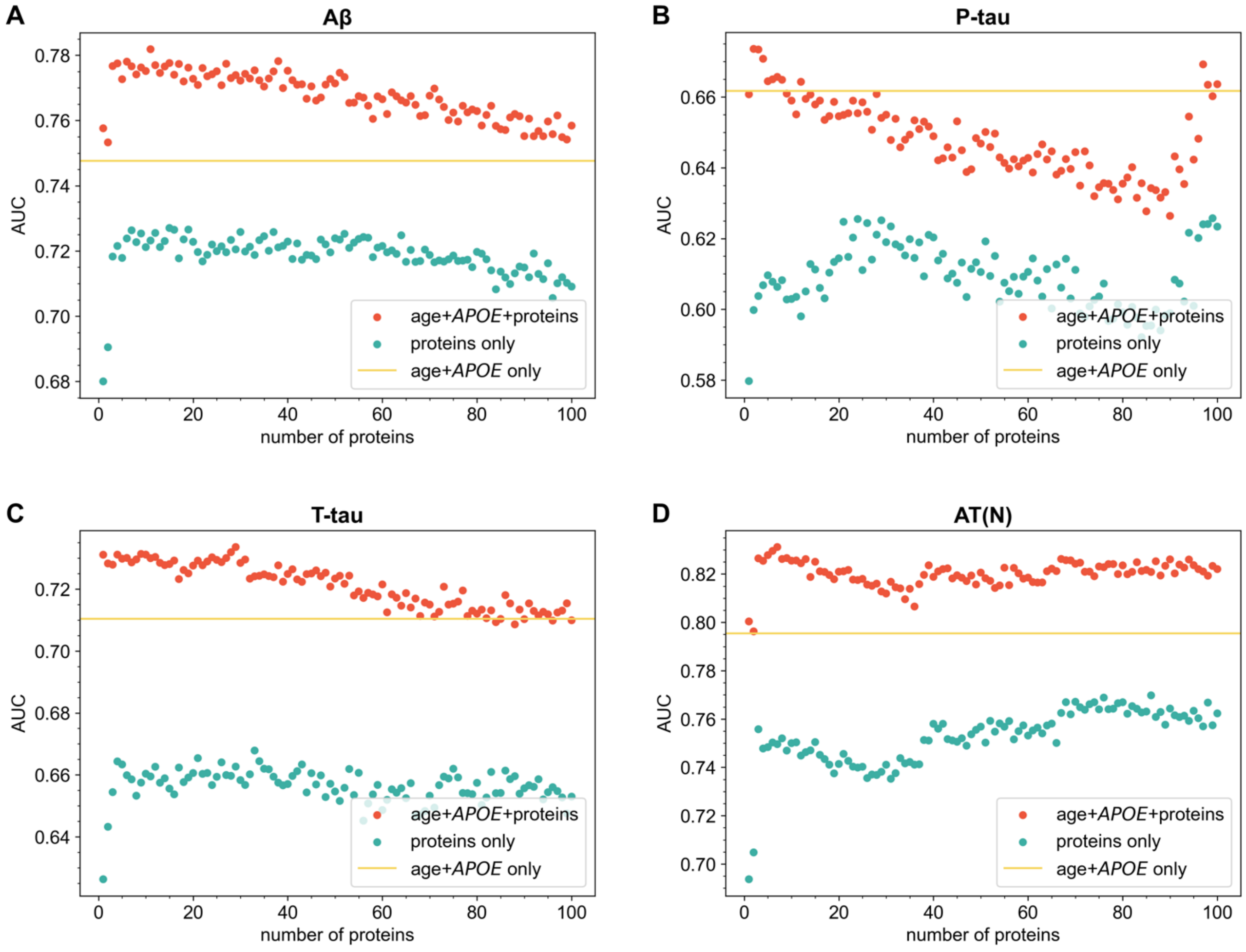

Supplement: Supplementary file 1 [file Data_Sheet_1.pdf]
